# From Gut Dysbiosis to Skin Inflammation in Atopic Dermatitis: Probiotics and the Gut–Skin Axis—Clinical Outcomes and Microbiome Implications

**DOI:** 10.3390/ijms27010365

**Published:** 2025-12-29

**Authors:** Adina Elena Micu, Ioana Adriana Popescu, Ioana Alina Halip, Mădălina Mocanu, Dan Vâță, Andreea Luana Hulubencu, Dragoș Florin Gheucă-Solovăstru, Laura Gheucă-Solovăstru

**Affiliations:** 1 Department of Dermatology, “Grigore T. Popa” University of Medicine and Pharmacy, 700115 Iași, Romania; adinaursache096@gmail.com (A.E.M.); alinaioanahalip@gmail.com (I.A.H.); drmadalinamocanu@yahoo.com (M.M.); danvata@gmail.com (D.V.); lsolovastru13@yahoo.com (L.G.-S.); 2 Department of Dermatology, “St. Spiridon” Emergency County Clinical Hospital, 700111 Iași, Romania; hulubencuandreea@gmail.com

**Keywords:** probiotics, gut–skin axis, dysbiosis, atopic dermatitis, microbiome

## Abstract

Atopic dermatitis (AD) is a chronic inflammatory skin disease in which barrier impairment, immune dysregulation, and gut–skin dysbiosis intersect, prompting growing interest in probiotics as microbiota-modulating adjuncts. We conducted a narrative review of peer-reviewed articles indexed in PubMed, Scopus, and Google Scholar, restricted to publications from 1 January 2018 to 31 October 2025 (searches last run in December 2025). Eligible evidence included randomized controlled trials (RCTs), observational studies, and mechanistic or conceptual reviews addressing microbiome alterations and microbiota-modulating interventions in AD. Most pediatric RCTs using multistrain, *Lactobacillus*-dominant formulations (often combined with *Bifidobacterium*) reported modest improvements in AD severity and pruritus and in selected barrier- and inflammation-related biomarkers. However, direct cutaneous microbiome “restoration” outcomes were reported in a minority of studies, and most clinical evidence relies on clinical endpoints and gut–skin axis plausibility rather than longitudinal skin microbiome readouts. Single-strain regimens showed inconsistent effects, and evidence in adolescents and adults remained heterogeneous. Mechanistically, probiotics may enhance short-chain fatty acid (SCFA) signaling, dampen toll-like receptor 2/4 (TLR2/4)-nuclear factor kappa B (NF-κB) activation, and promote interleukin-10 (IL-10)- and transforming growth factor-β (TGF-β)-driven tolerance. Probiotics are a biologically plausible adjunct targeting the gut–skin axis in AD and are generally well tolerated; however, heterogeneity across trials, limited follow-up, inconsistent adverse-event reporting, and scarce skin microbiome endpoints preclude firm clinical recommendations.

## 1. Introduction and Background

### 1.1. The Skin and Gut Microbiome

The skin and gut are both active, complex immunological and neuroendocrine organs that are in constant contact with the external environment and host diverse microbial communities. Proper functioning of these systems is essential for maintaining homeostasis and supporting survival. The skin, as the body’s largest organ, acts as a protective barrier against injury and microbial invasion, while the gut harbors trillions of microorganisms, functioning as a “virtual organ” closely linked to the host’s health and longevity. The gut microbiome exerts both beneficial and detrimental effects on the physiology and homeostasis of gut and skin tissues [1].

Traditionally, the skin has been viewed as a protective barrier against physical, chemical, and biological insults. Advances in metagenomic research, however, have shown that nearly every human body site, including the skin, harbors diverse microbial communities.

Colonized by bacteria, fungi, and viruses, the skin is not merely a passive shield but a dynamic ecosystem, shaped by mutual adaptation with the host and influenced by environmental and nutritional factors. Similar to other body sites, the skin microbiota plays a vital role in maintaining normal skin physiology, while dysbiosis has been linked to various skin diseases. Understanding and characterizing the skin microbiome can provide valuable insights into these conditions and help unravel the complex interactions between humans and their resident microbes [2].

On the other hand, the human gut microbiota is a complex ecosystem containing trillions of microorganisms—bacteria, viruses, fungi, and other microbes—that have coevolved with humans, influencing and being influenced by our physiology, diet, and lifestyle. This diverse community plays essential roles in gut function, with its homeostasis being fundamental to overall health. Balanced gut microbiota regulates intestinal inflammation, supports metabolic stability, and guides the maturation and modulation of the immune system. Disruption of this balance, known as dysbiosis, has been associated with numerous conditions, including inflammatory bowel disease, obesity, type 2 diabetes, and inflammatory skin diseases such as atopic dermatitis (AD), psoriasis, and acne [3]. However, reported dysbiosis signatures vary substantially across diseases and even across cohorts within the same disease, depending on host factors (age, diet, medications), sampling site, sequencing platform, and bioinformatic pipelines; therefore, taxon-level patterns should be interpreted as context-dependent trends rather than universal “signatures.” Accordingly, throughout this review we avoid extrapolating specific taxonomic shifts across conditions unless directly supported by disease-specific evidence. For example, in moderate-to-severe acne, gut microbiota alterations have been reported to include an increased relative abundance of *Proteobacteria/Enterobacteriaceae* and decreased *Bifidobacterium* spp. compared with controls in some cohorts [4].

### 1.2. The Gut–Skin Axis and Neuro-Immuno-Cutaneous-Endocrine (NICE) Network

**Figure 1:**
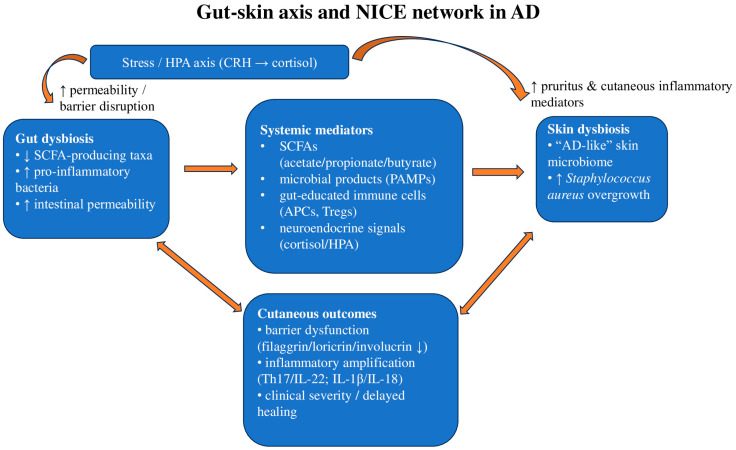
Mechanistic overview of gut–skin axis/NICE network signaling in AD: gut dysbiosis has been linked to altered circulating metabolites, immune trafficking, and neuroendocrine mediators (stress/HPA axis), may contribute to skin dysbiosis and cutaneous inflammatory outcomes. SCFAs, short-chain fatty acids; FFAR, free fatty acid receptor; HDAC, histone deacetylase; AHR, aryl hydrocarbon receptor; PAMPs, pathogen-associated molecular patterns; APCs, antigen-presenting cells; Tregs, regulatory T cells; CRH, corticotropin-releasing hormone; HPA, hypothalamic–pituitary–adrenal; NLRP3, NLR family pyrin domain containing 3. Arrows indicate direction of effect; bidirectional arrows indicate reciprocal interactions.

The gut–skin axis describes the bidirectional communication between gut dysbiosis and skin homeostasis (Figure 1). Both the gut and skin fulfill important immunological and neuroendocrine roles, constantly adapting to environmental changes to maintain balance. Each organ harbors a distinct microbiome that is thought to support local immune homeostasis, while systemic circulation enables cross-talk via immune cells, hormones, and microbial metabolites. Accordingly, dysbiosis in one site may influence the other, potentially contributing to immune dysregulation and impaired barrier defense [1].

Park and colleagues reported that, in AD, intestinal alterations—especially the loss of short-chain fatty acids (SCFAs)-producing taxa and the relative expansion of pro-inflammatory bacteria—were accompanied by a more ‘AD-like’ skin microbiome, findings that are consistent with (but do not prove) a shared gut–skin pathway. They propose that circulating microbial metabolites (notably SCFAs), together with gut-educated immune cells (Regulatory T cells (Tregs) and antigen-presenting cells), may reach the skin and could contribute to modulation of cutaneous immune responses. A downstream reduction in *Staphylococcus aureus* burden has been hypothesized, but direct causal evidence in humans remains limited. This immune-metabolic signaling is further shaped by stress-related neuroendocrine mediators (e.g., cortisol), which supports the concept of a neuro-immuno-cutaneous network linking microbiome imbalance to clinical severity [5].

SCFAs exert immunoregulatory effects through both receptor-dependent and receptor-independent pathways. Recent evidence indicates that acetate, propionate, and particularly butyrate can signal via the free fatty acid receptors FFAR2/GPR43 and FFAR3/GPR41 expressed on intestinal epithelial and antigen-presenting cells, and these pathways have been associated with reduced pro-inflammatory cytokine production and improved barrier integrity in experimental and translational settings [6]. In parallel, SCFAs may enter immune cells and inhibit histone deacetylases (HDACs), an epigenetic mechanism that has been linked to the differentiation and stabilization of Forkhead box P3 (FOXP3^+)^ regulatory T cells and may support peripheral tolerance [7]. In addition, reduced availability of microbiota-derived tryptophan metabolites has been proposed to weaken aryl hydrocarbon receptor (AHR)-dependent barrier-protective programs in keratinocytes, which can counteract IL-4/IL-13-mediated downregulation of filaggrin, loricrin, and involucrin [8]. Taken together, G protein-coupled receptor (GPCR) engagement and HDAC inhibition offer biologically plausible mechanisms by which microbial fermentation products could contribute to systemic immune regulation, supporting the rationale for the gut–skin axis in inflammatory dermatoses while acknowledging that direct causal links in humans remain to be fully established.

Moreover, Patel et al. emphasize that dendritic cells and lymphocytes activated within gut-associated lymphoid tissue (GALT) and mucosa-associated lymphoid tissue (MALT) after exposure to microbial antigens can recirculate and reprogram cutaneous immune responses, including IgA production and antimicrobial peptide expression. They also link microbial metabolites—SCFAs and tryptophan-derived AHR ligands—to epithelial repair and inflammation control. Gut-derived pathogen-associated molecular patterns (PAMPs) and *Staphylococcus aureus* products have been reported to activate the NLR family pyrin domain containing 3 (NLRP3) inflammasome in experimental models, which can be accompanied by IL-1β and IL-18 release and enhanced Th17/IL-22 signaling; however, the extent to which this pathway drives clinical AD in humans remains to be fully established [9]. Because these mediators are themselves modulated by the hypothalamic–pituitary–adrenal (HPA) axis (cortisol) and by neurovegetative tone, the authors place gut–skin signaling within a broader NICE framework that conceptualizes how intestinal dysbiosis may contribute to chronic skin inflammation or delayed lesion healing [10].

Additionally, intestinal dysbiosis may contribute to cutaneous inflammation and impair neuropsychological homeostasis. Psychological stress has been associated with intestinal barrier dysfunction and exacerbation of inflammatory skin disease. Stress-related activation of the hypothalamic–pituitary–adrenal (HPA) axis may increase corticotropin-releasing hormone (CRH)/cortisol signaling; CRH receptors on mast cells and keratinocytes may promote TNF-α, IL-6, and pruritogenic mediators in the skin, while stress-related pathways have also been linked to increased intestinal permeability, potentially facilitating systemic exposure to microbial products [11,12]. These processes may interact with and amplify the type-2-skewed inflammatory milieu initiated by dysbiosis [13,14].

At birth, human skin is colonized by a diverse community of microorganisms (bacteria, fungi, and viruses), and several studies suggest that the initial microbial profile may differ by mode of delivery, body site, and early-life exposures [15,16]. Vaginal delivery has been associated with early enrichment of vaginal-associated taxa (including *Lactobacillus* spp.), whereas cesarean delivery has been linked to relatively greater contributions from skin- and environment-associated taxa (e.g., *Staphylococcus* spp., *Corynebacterium* spp., and *Cutibacterium* spp.; formerly *Propionibacterium* [17]) [15,16,18]. However, reported patterns are heterogeneous and can be influenced by factors such as perinatal antibiotic exposure, feeding, hospitalization, and sampling methodology [15,16]. Early commensal colonization is thought to contribute to barrier maturation and immune education, but direct causal links between specific neonatal taxa and later AD phenotypes remain to be established [15]. Certain commensal staphylococci have been proposed to play protective roles in a strain-dependent manner, whereas other strains may induce pro-inflammatory signaling, underscoring the importance of strain-level resolution in future studies [19,20].

### 1.3. Atopic Dermatitis

Atopic dermatitis (AD) is a chronic, relapsing inflammatory dermatosis marked by xerosis, eczematous lesions, and often debilitating pruritus. Its prevalence continues to rise in industrialized settings, affecting an estimated 15–30% of children and around 10% of adults worldwide. Beyond the cutaneous symptoms, AD represents the entry point into the atopic march: children with early or persistent AD have a substantially higher risk of subsequently developing IgE-mediated food allergy, allergic rhinitis, and asthma/bronchial hyperreactivity, underscoring the systemic nature of the disease. Recurrent scratching driven by severe pruritus can further compromise the epidermal barrier and is commonly associated with increased *Staphylococcus aureus* abundance, potentially amplifying immune dysregulation and contributing to a self-reinforcing cycle between inflammation and cutaneous dysbiosis [21,22,23].

AD is characterized by a primary epidermal barrier defect involving diminished or dysfunctional filaggrin, altered ceramide profiles, and increased transepidermal water loss (TEWL), which facilitates penetration of allergens and microbial products. Filaggrin insufficiency may shift stratum corneum pH toward neutrality, potentially favoring *Staphylococcus aureus* overgrowth and reducing antimicrobial peptide (AMP) activity. These barrier alterations intersect with type 2-skewed immunity (IL-4/IL-13/IL-31), which further downregulates filaggrin, loricrin, and involucrin, creating a self-reinforcing loop between structural damage and dysbiosis, as detailed by Yang et al. [24].

### 1.4. AD and the Cutaneous and Gut Microbiome

Since the early descriptions of host–microbe symbiosis, the gut microbiome has come to be recognized as a dynamic immunologic organ that continuously communicates with intestinal immune cells. Alterations in its composition have been linked to multiple allergic and inflammatory conditions, including AD. The gut microbiome is increasingly viewed as an important contributor to gut–skin signaling, and accumulating data suggest that shifts in gut communities may influence AD risk and clinical expression. Clarifying how extracutaneous organs, especially the intestine, shape cutaneous inflammation may therefore improve disease control and patients’ quality of life [1,21,25].

Moreover, a prospective age- and sex-matched case–control study in adults with dermatologist-verified AD reported poorer oral health indices and oral dysbiosis compared with controls, alongside significantly different oral microbiome diversity and disease-associated taxa profiles [26]. AD provides a useful model for exploring how cutaneous and intestinal microbial communities may be perturbed in parallel and potentially interact with host inflammation. On the skin, barrier disruption together with type 2-skewed immunity is commonly associated with increased *Staphylococcus aureus* abundance and reduced microbial diversity, patterns that have been reported to track with clinical severity in many cohorts [27,28]. In parallel, several studies have described gut microbiome alterations in patients with AD and immune profiles consistent with systemic activation (e.g., Th2/Th17 skewing and reduced tolerance), which could plausibly influence cutaneous inflammatory responsiveness and barrier vulnerability; however, causal directionality and mediation remain incompletely established [21,23]. Importantly, children and adults do not share the same cutaneous ecology: differences in sebum levels, pH, lipid composition, and barrier maturation across early life shape distinct microbial communities and may contribute to age-dependent variability in disease stability and treatment response [27,28]. This variability should be considered when designing and interpreting microbiota-modulating interventions in AD [21,23,27,28,29].

### 1.5. Current Standard of Care and Place of Microbiome-Targeted Adjuncts

Conventional pharmacological management of AD is centered on barrier repair and anti-inflammatory control. Topical corticosteroids remain first-line for acute flares, with potency tailored to age, anatomical site, and disease severity [30,31]. Non-steroidal topical immune modulators, including topical calcineurin inhibitors (tacrolimus, pimecrolimus) and other steroid-sparing anti-inflammatory options (e.g., topical PDE4 inhibition and topical JAK inhibition, where approved), are commonly used for sensitive areas and maintenance strategies [30,31]. For moderate-to-severe or refractory disease, escalation may include phototherapy and systemic/targeted therapies, including biologics and oral JAK inhibitors, according to patient profile and risk–benefit assessment [32].

In this therapeutic landscape, microbiome-targeted approaches (probiotics/prebiotics/synbiotics/postbiotics) should be regarded as adjunctive strategies that may complement rather than replace established pharmacologic treatment, and trial outcomes should be interpreted in the context of concomitant topical/systemic therapy [27,28].

### 1.6. Probiotics

Probiotics, together with selectively fermented prebiotics, are being explored as adjunct tools to modulate host–microbiome interactions in Dermatology. Their relevance in AD stems from the ability to influence both intestinal and cutaneous immunity and to work alongside standard topical therapy [21,33].

Beyond dermatologic indications, probiotic supplementation has been investigated in allergic disorders, Irritable Bowel Syndrome (IBS)/Inflammatory Bowel Disease (IBD), infection-related diarrhea, and certain metabolic disturbances, although reported benefits are heterogeneous and depend on the strain(s) used, the population studied, and the outcomes assessed [6,34]. Proposed mechanisms include modulation of gut microbial composition and immune signaling rather than uniformly demonstrated “reshaping” effects across conditions [6,34].

Mechanistically, probiotic actions can be conceptualized into four complementary pathways. First, probiotics may competitively exclude pathogens by occupying mucosal binding sites and competing for nutrients [7,35]. Second, they can produce antimicrobial metabolites, such as SCFAs, bacteriocins, and hydrogen peroxide (H_2_O_2_), which help limit the overgrowth of potentially harmful taxa [7,35]. Third, probiotics may reinforce epithelial barrier function by increasing mucus production and enhancing tight-junction protein expression, thereby reducing microbial translocation [7,35]. Finally, probiotics may exert immune and neuroendocrine modulatory effects by influencing dendritic-cell maturation and T-cell responses, as well as microbially driven neurotransmitters (e.g., serotonin, dopamine, and GABA) along the gut–brain–skin axis [7,35].

### 1.7. Probiotics and AD

Recent literature suggests a central involvement of the gut–skin axis in AD, in which intestinal and cutaneous dysbiosis may act together to impair barrier function and skew immune responses. Several meta-analyses and narrative/scoping reviews report that probiotic, prebiotic, or synbiotic regimens, particularly multistrain formulations enriched in *Lactobacillus* spp., have been associated with a lower AD incidence in high-risk infants and with reduced disease severity (improved SCORAD/EASI scores), whereas *Bifidobacterium*-only preparations show more variable results. Mechanistically, probiotics may modulate microbial community composition and function, promote tolerance toward commensals, and exert anti-inflammatory and antioxidant effects, potentially counteracting dysbiosis-related immune-barrier perturbations along the gut–skin axis. Although most data derive from pediatric cohorts, more recent studies suggest that microbiota-modulating interventions may also benefit adolescents and adults with AD [21,23,29,36].

### 1.8. The Mechanism of Probiotics in Improving Skin Diseases

**Figure 2:**
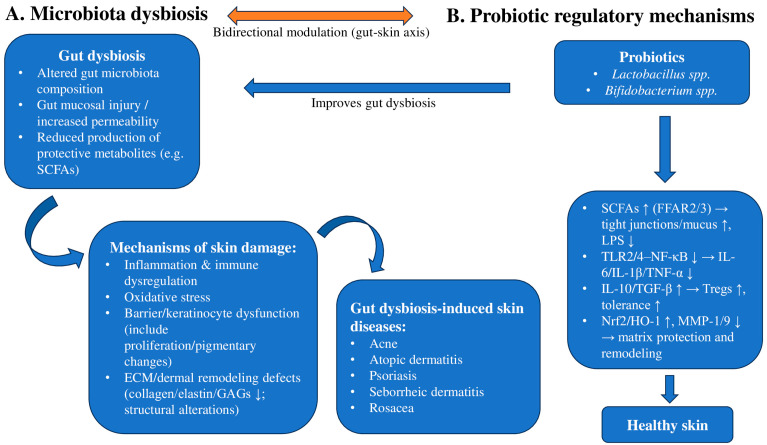
Two-column schematic of the gut–skin axis highlighting bidirectional modulation between (**A**) microbiota dysbiosis and (**B**) probiotic regulatory mechanisms. Dysbiosis is associated with impaired barrier integrity and inflammatory/immune dysregulation, while probiotics (e.g., *Lactobacillus* spp., *Bifidobacterium* spp.) may modulate gut microbial function and systemic immune signaling, with potential downstream relevance for skin inflammation. SCFAs, short-chain fatty acids; FFAR2/FFAR3, free fatty acid receptors 2/3; LPS, lipopolysaccharide; TLR, Toll-like receptor; NF-κB, nuclear factor kappa B; Tregs, regulatory T cells; Nrf2, nuclear factor erythroid 2-related factor 2; HO-1, heme oxygenase-1; MMP, matrix metalloproteinase. Arrows indicate direction of effect; bidirectional arrows indicate reciprocal interactions.

Probiotics such as *Lactobacillus* and *Bifidobacterium* have been reported in some studies to modulate intestinal microbial balance and gut–skin axis-related immune pathways, with potential downstream relevance for skin inflammation. By increasing SCFAs production and engaging FFAR2/FFAR3 signaling on epithelial and immune cells, probiotics may support tight-junction integrity, promote mucus layer function, and attenuate markers consistent with reduced lipopolysaccharide (LPS) translocation. These effects may limit systemic inflammatory signals that would otherwise aggravate skin lesions [6,35]. At the immune level, probiotics may rebalance immune responses by downregulating TLR2/TLR4-NF-κB signaling and decreasing pro-inflammatory cytokines, including IL-6, IL-1β, and TNF-α. In parallel, they may promote regulatory mediators such as IL-10 and TGF-β, supporting regulatory T-cell responses and improved tolerance to skin commensals, which is particularly relevant for AD, and potentially relevant for other inflammatory dermatoses [7,21]. In addition, several strains (e.g., *L. rhamnosus*, *L. plantarum*) have been reported to activate antioxidant pathways (Nrf2/HO-1) and inhibit UV- or inflammation-induced MMP-1/MMP-9. This may help preserve collagen, elastin, and glycosaminoglycans and support dermal matrix remodeling, with potential implications for photoaging, wound healing, and rosacea flares (Figure 2) [37,38]. Although such mechanistic observations are consistent with clinical findings in AD, the strongest clinical evidence supporting probiotics derives from AD trials and meta-analyses, whereas data in other inflammatory dermatoses (e.g., psoriasis) are more heterogeneous and limited. Overall, multi-strain, *Lactobacillus*-based formulations used as adjuncts to standard therapy have been reported to be associated with modest improvements in disease severity scores and selected barrier-related markers in some studies, but evidence outside AD remains inconsistent and should be interpreted cautiously [23,33].

Therefore, this narrative review summarizes current evidence on gut- and skin-microbiome alterations in AD, the molecular mechanisms linking dysbiosis to cutaneous inflammation (including the NICE network), and the biological rationale and available clinical data for probiotic, prebiotic, and synbiotic interventions.

Direct evidence of skin microbiome restoration is limited; most studies report clinical outcomes.

This narrative review aims to critically synthesize clinical and mechanistic evidence linking the gut–skin axis to AD and to appraise microbiota-modulating interventions (probiotics, prebiotics, and synbiotics), providing an evidence-based perspective on their potential role and current limitations.

## 2. Evidence Synthesis

### 2.1. Evidence of Gut and Skin Dysbiosis in AD

Multiple clinical and microbiome studies have reported that patients with AD (both children and adults) may display reduced gut microbial diversity and compositional shifts compared with healthy controls [5,21,23,39]. In several cohorts, these shifts have included a higher relative abundance of *Proteobacteria/Enterobacteriaceae* and lower levels of *Bifidobacterium* and *Lactobacillus*, although findings vary across populations, disease endotypes, and analytical methods [5,21,39]. Some studies have further reported associations between gut dysbiosis features, higher disease activity, and increased cutaneous *Staphylococcus aureus* burden, which is consistent with the possibility of coordinated gut–skin microbial and immune alterations in AD, although causality and directionality cannot be inferred from the available observational evidence [5,21,23].

### 2.2. Early-Life Colonization and Later AD Phenotypes

Evidence from neonatal and infant cohorts suggests that mode of delivery and early postnatal microbial colonization may be associated with differences in early cutaneous microbial profiles, although findings are heterogeneous and strongly shaped by perinatal exposures (e.g., intrapartum antibiotics, feeding practices, hospitalization/NICU environment) and sampling/analytical approaches. In some cohorts, vaginal delivery has been linked to a higher early representation of vaginal-associated taxa (including *Lactobacilli*), whereas cesarean delivery has been associated with relatively greater contributions of skin- and environment-associated microbes and, in certain studies, lower early diversity; however, these patterns are not consistent across populations or timepoints [28,40]. Early-life colonization has also been associated with immune “education” signals, including markers related to FOXP3^+^ regulatory T-cell responses, but the evidence is largely observational and does not establish causality or long-term effects on later AD risk [40].

### 2.3. Probiotic/Prebiotic/Synbiotic Interventions in AD

Recent umbrella meta-analyses and systematic reviews indicate that multi-strain preparations dominated by *Lactobacillus* spp. (often combined with *Bifidobacterium* and, in some trials, with prebiotic substrates as synbiotics) are associated with modest but clinically relevant reductions in SCORAD/EASI and fewer flares, particularly in pediatric AD; in contrast, single-strain *Bifidobacterium* products and prebiotics alone show more inconsistent or smaller effects [29,33,36,41,42]. In a 2025 umbrella meta-analysis, synbiotics and multi-strain/*Lactobacillus*-driven interventions significantly decreased SCORAD, whereas *Bifidobacterium*-only and prebiotics-only interventions did not show a significant effect on severity [43]. Similarly, the 2024 EAACI task force meta-analysis reported a significant reduction in SCORAD across RCTs assessing probiotics alone or combined with prebiotics [42]. These trials also reported improvements in patient-/parent-reported outcomes (e.g., itch and sleep), supporting a real, although moderate benefit, plausibly linked to gut–skin axis and microbiome-related immunomodulatory mechanisms [33,44].

### 2.4. Mechanistic/Proof-of-Concept Studies

Mechanistic pathways described below are derived mainly from experimental and translational evidence and should be interpreted as plausible, hypothesis-generating mechanisms rather than established causal chains in AD. Probiotics may increase SCFAs production and engage SCFA-sensing pathways (e.g., FFAR2/GPR43 and FFAR3/GPR41) on epithelial and immune cells, which is consistent with improved barrier function and attenuation of markers suggestive of reduced lipopolysaccharide (LPS) translocation [6,7]. In experimental settings, probiotic-related immunomodulation has also been reported to influence innate immune signaling (including TLR-NF-κB-related pathways) and cytokine profiles, with lower pro-inflammatory mediators (e.g., IL-6, IL-1β, TNF-α) and higher anti-inflammatory signals (e.g., IL-10, TGF-β), alongside effects on regulatory T-cell-associated responses [6,7]. In addition, antioxidant and barrier-regulatory programs (including NRF2-linked pathways) and extracellular-matrix-related remodeling processes have been discussed as potentially relevant to tissue resilience, although direct causal mediation of these pathways in human AD remains incompletely established [8,37]. Overall, these mechanistic observations provide biological plausibility linking gut microbiome modulation to skin-relevant immune and barrier pathways, while definitive causal links to clinical outcomes in human AD have not been proven.

### 2.5. Other Extracutaneous Microbiomes

A recent case–control study reported that adults with AD may have impaired oral health and oral dysbiosis, with differences in oral microbiome diversity and taxonomic profiles compared with controls, highlighting an additional microbial niche potentially relevant to AD [26]. This supports an expanded “skin–gut–oral” model in AD.

### 2.6. Clinical Evidence Summary (RCTs)

To provide an at-a-glance overview of the clinical evidence, we summarized randomized controlled trials (RCTs) evaluating microbiome-targeted interventions in AD identified through database searches and reference screening (Table 1). All trial characteristics reported in Table 1 (formulation/strain(s), dose and dosing frequency, duration, population, comparator, and outcomes) were extracted exclusively from the primary RCT publications and, when available, their Supplementary Materials. Secondary sources (e.g., reviews/meta-analyses) were used only to help identify potentially eligible trials, not as a source for data extraction. When a specific intervention detail could not be verified from the primary report and/or its supplements, the corresponding field was labeled “NR”.

Overall, the RCT evidence base is heterogeneous with respect to participant age (pediatric and adult cohorts), baseline severity, background therapy, intervention composition (single-strain vs. multi-strain probiotics and synbiotics, as well as one non-viable preparation dosed in grams/day), and outcome selection. Most trials assessed clinical severity using SCORAD (and less consistently EASI), with several also reporting flare-related outcomes and/or topical corticosteroid use. Across trials, results were mixed: some RCTs reported statistically significant improvements in severity scores and/or steroid-sparing effects compared with placebo, whereas others found no meaningful between-group differences across timepoints or responder thresholds. Immunologic endpoints, when reported, were variably selected and did not consistently parallel clinical response. Given between-trial heterogeneity and occasional incomplete reporting of intervention granularity, inference should focus on the direction and consistency of effects across well-described trials rather than on strain- or dose-specific claims from single studies; accordingly, we synthesize the RCT evidence narratively and interpret findings in the context of reporting quality and clinical comparability.

## 3. Clinical Implications, Limitations, and Future Directions

### 3.1. Lactobacillus and Bifidobacterium as Dermatologic Probiotics

*Lactobacillus* is among the best-characterized lactic acid-producing genera explored for skin-related applications. In experimental models, specific strains have been reported to attenuate pro-inflammatory signaling in keratinocytes, dampen substance P-associated responses, and support aspects of barrier repair. In vivo and translational studies further suggest that *L. acidophilus* may reduce UV-associated MMP induction and wrinkle formation, *L. johnsonii* has been associated with faster immune recovery following UV-induced immunosuppression, and *L. rhamnosus* has been explored in acne, with proposed effects on insulin-related pathways reported in limited studies [21,37]. *Bifidobacterium* may exert complementary barrier-related effects; for example, *B. breve* has been reported to reduce transepidermal water loss (TEWL), dryness, and structural damage in some studies, while mixed *Lactobacillus–Bifidobacterium* formulations have been associated with reduced food sensitization and AD in pediatric cohorts [23,36].

### 3.2. Early-Life Microbiome, Hygiene Concept, and AD Risk

Consistent with the modern “microbial exposure” version of the hygiene hypothesis, reduced microbial diversity in early life and a gut microbiome poor in bifidobacterial have been associated with earlier AD onset, more persistent phenotypes, and higher rates of comorbid allergy/asthma [1,40]. Together, these observations support the view that AD may involve systemic microbiome-immune interactions, rather than being purely skin-limited.

### 3.3. Breadth of Probiotic Effects

Recent randomized trials and meta-analyses suggest that selected strains and combinations may provide clinical benefits in AD and, in some analyses, other inflammatory conditions, although effect sizes and consistency vary by strain, population, and outcome [33,35]. These findings support the concept that dermatologic outcomes may reflect broader microbiota-immune crosstalk rather than uniform effects across diseases.

### 3.4. Strain Specificity and Colonization

A key point from newer studies is that colonization is strain-, host-, and diet-dependent; some *Lactobacillus/Bifidobacterium* strains can persist and keep modulating immunity, others are transient. That may be relevant in dermatology, as durable colonization has been proposed as one factor that could contribute to sustained SCORAD/EASI improvement in some trials, whereas other studies report more transient or variable effects [6].

### 3.5. Gut–Skin Axis in Chronic Inflammatory Dermatoses

Dysbiosis-driven immune activation and barrier impairment have been described not only in AD but also in psoriasis and hidradenitis suppurativa, supporting continued investigation of microbiome-directed strategies as adjunctive approaches [38,55]. Accordingly, probiotics, prebiotics, synbiotics, and postbiotics may be considered potential adjuncts—not replacements—to standard dermatologic regimens.

### 3.6. Toward Personalized/Profiling-Based Use

Because not all strains have the same immunologic signature—some *Lactobacillus* strains can even enhance allergic responses in children—interventions may need to be tailored to the patient’s microbiome and disease phenotype; this is the rationale for microbiome-guided/personalized cosmetics and supplements [21,28].

### 3.7. Timing and Maternal Supplementation

Several trials indicate that perinatal (prenatal + early postnatal) probiotic exposure, especially via maternal supplementation and transfer through breast milk, provides better protection against AD than delayed or prolonged (>12 months) regimens. Timing, strain mix, and host factors all modulate efficacy, which explains the heterogeneity seen across studies [33,34].

### 3.8. Strength of Evidence

Recent umbrella reviews pooling >100 meta-analyses conclude that multi-strain, *Lactobacillus*-dominant products are the most consistently protective and appear safe in pediatrics, but the certainty of evidence is still “weak to suggestive”, mainly because of heterogeneous populations, doses, and outcomes. Larger, strain-defined RCTs are still needed [33].

In clinical settings, microbiome-directed interventions are generally discussed in the literature as potential adjuncts to standard AD care rather than stand-alone therapies. Across the available RCTs in mild-to-moderate AD (many in pediatric cohorts), multi-strain formulations, often *Lactobacillus*-dominant and sometimes combined with *Bifidobacterium*, have been the most frequently studied, with variable effects across outcomes and populations [35,55]. In terms of duration, published trials typically used administration periods of approximately 4–12 weeks; evidence for shorter courses is limited, and shorter interventions have not consistently shown benefit in the available reports.

For prevention, some trials in high-risk infants suggest that perinatal exposure (during pregnancy and/or lactation), followed by early postnatal supplementation, may be associated with more consistent reductions in AD incidence than later supplementation; however, the overall certainty remains limited by heterogeneity in strains, timing, and study designs [39]. Reported effects also appear to differ by baseline risk and early-life exposures (e.g., delivery mode, family history), but current evidence does not allow firm prioritization of specific subgroups. Safety and tolerability have generally been reported as acceptable in the included studies, yet follow-up duration and adverse-event ascertainment vary and long-term safety data, particularly in vulnerable populations, remain limited.

Furthermore, product choice may be informed by strain-level transparency and by the patient’s barrier status. Preparations that specify strain, CFU, and duration are preferable to generic “probiotic” products, and outcomes can be monitored with simple clinical markers—disease severity (SCORAD/EASI), TEWL, and *S. aureus* colonization, where available—to document response and to avoid unnecessary long-term supplementation.

Future research should prioritize integrated, patient-level studies that profile the gut and skin microbiome in parallel, in the same subjects, to document true bidirectional gut–skin crosstalk. These microbial data need to be linked to clinical severity scores (SCORAD, EASI) and to systemic metabolic/inflammatory markers that may reflect dysbiosis, so we can identify microbiome signatures that actually matter clinically.

Comparative trials should also directly test probiotics vs. postbiotics vs. synbiotics, since postbiotics may deliver more reproducible, host-targeted effects and avoid colonization variability. Because AD is heterogeneous, future studies must use age- and phenotype-specific regimens (pediatric vs. adult AD; intrinsic vs. extrinsic AD) instead of one-size-fits-all protocols.

Finally, upcoming trials should adopt minimum reporting standards—strain, CFU/dose, duration, co-interventions—and should include long-term safety and durability assessments, especially for infants and pregnant/lactating women.

### 3.9. Skin-Targeted Implications

Cutaneous dysbiosis in AD has commonly been reported to involve reduced microbial diversity, depletion of commensal staphylococci, and increased *Staphylococcus aureus* abundance, supporting interest in adjunctive, skin-directed strategies alongside oral microbiota-modulating approaches. Hrestak et al. described AD-associated skin microbial patterns and discussed restoration of commensal communities as a potential therapeutic objective [44]. Smythe and Wilkinson likewise highlighted that topical products designed to be microbiome-friendly may help normalize skin pH, support antimicrobial peptide (AMP) activity, and reduce conditions that favor pathogen expansion [56]. Taken together, these observations provide a rationale for considering emollients enriched with pre-/pro-/postbiotics as a local complement to systemic modulation, while acknowledging that clinical and microbiome endpoints remain heterogeneous across studies.

### 3.10. Targeting Staphylococcus aureus

Because AD severity often parallels *S. aureus* burden, strategies that reduce colonization can potentiate microbiome-supportive topicals. Li et al. showed, at the transcriptomic level, that *S. aureus* adapts when shifting from nasal to lesional AD skin, expressing virulence programs relevant for inflammation [57]. This provides molecular justification for decolonization protocols (bleach baths, intranasal mupirocin) and for topical formulations that favor recolonization with protective staphylococci.

### 3.11. Linking Gut–Skin Correction

Park et al. documented that AD can display parallel alterations in gut and skin microbiota, suggesting that systemic (oral) and local (topical) measures may be combined rather than used in isolation [5]. More recently, Wrześniewska et al. emphasized that therapeutic strategies may aim both at reducing dysbiosis and at restoring barrier-immune crosstalk [58]. Finally, even experimental data on gut–skin enhancement with probiotic-containing formulations show that dermal cells can respond to microbially derived metabolites, reinforcing the biological plausibility of pairing oral probiotics with skin-directed care [59].

## 4. Literature Search Strategy

### 4.1. RCT-Focused Search for Clinical Evidence

To enhance transparency and reproducibility of the clinical evidence base, we conducted a focused search to identify randomized controlled trials (RCTs) of microbiome-targeted interventions in AD for the clinical evidence summary (Table 1). PubMed and Scopus were searched using predefined Boolean strategies combining AD terms with gut–skin axis/microbiome concepts and intervention-related keywords (e.g., probiotic*, synbiotic*, prebiotic*, postbiotic*, microbiome modulation). Google Scholar was searched using targeted keyword combinations. Searches were last run in December 2025; however, retrieved records were filtered by publication/indexing date to the predefined evidence cutoff (31 October 2025) to ensure a consistent and verifiable evidence base for synthesis, and records published after this date were not considered. Full database-specific search strings, limits, and retrieval counts are provided in Table S1, and the study selection workflow is summarized in a PRISMA-style flow diagram (Figure S1). For Google Scholar, results were sorted by relevance and the first 100 records within the coverage window were screened, with 14 items retained for closer inspection.

Eligibility criteria for inclusion in Table 1 were specified a priori using a PICO framework: participants with clinician-diagnosed AD; microbiome-targeted interventions (probiotic, prebiotic, synbiotic, or postbiotic regimens) compared with placebo and/or standard care/control; and reporting at least one AD-relevant clinical endpoint. Outcomes of interest included validated disease severity measures (SCORAD and/or EASI, when available) as primary clinical endpoints, with secondary outcomes encompassing pruritus, patient-reported outcomes (e.g., quality of life), and safety/tolerability and adverse events. Only English-language RCTs with a control/comparator arm and sufficient intervention/outcome reporting to permit extraction were eligible for Table 1.

The database search retrieved 157 records from PubMed and 322 from Scopus. After removal of duplicates across sources (n = 39), 440 unique records underwent title/abstract screening. Twelve reports were assessed in full text, and 10 RCTs met eligibility criteria and were included in Table 1. Full-text exclusions were primarily due to non-randomized design or absence of a control/placebo comparator, lack of AD-relevant clinical outcomes, insufficient intervention reporting or unusable data (e.g., conference abstracts only), non-English language, or duplicate/overlapping reports. Data extraction for Table 1 was performed exclusively from the primary RCT reports (and Supplementary Materials when available); secondary sources were used only to identify potentially eligible trials.

### 4.2. Narrative Search for Mechanistic and Observational Evidence

To inform the mechanistic and observational sections of the manuscript, we additionally conducted a narrative, targeted search of peer-reviewed literature on the gut–skin axis, dysbiosis patterns, immune-barrier pathways, and microbiome-related concepts relevant to AD. This component relied on targeted keyword searches, review-led “citation chasing” (screening reference lists of eligible reviews and primary studies), and inclusion of key observational human studies, guidelines, and selected experimental literature used to support mechanistic interpretation. Animal and in vitro studies were used selectively for mechanistic context but were not included in the clinical RCT evidence synthesis (Table 1). Studies were excluded if they were non-English, duplicates, conference abstracts without full data, or focused exclusively on gastrointestinal disease without dermatologic relevance.

Given substantial heterogeneity in study designs, populations, interventions (strains/formulations, dosing, duration), and outcome measures, a quantitative meta-analysis was not pursued; evidence was synthesized narratively and organized into mechanistic and clinical sections. In line with the narrative review design, no protocol was registered; however, the scope, eligibility criteria, outcomes of interest, and synthesis approach were defined a priori and are described in Section 4. To enhance transparency and reduce susceptibility to selective reporting, the clinical RCT evidence base was compiled using a focused, reproducible search strategy (Table S1) and data were extracted exclusively from primary RCT reports (and Supplementary Materials when available).

During manuscript preparation, ChatGPT (OpenAI, San Francisco, CA, USA-model: GPT-5.2 Thinking) was used solely to assist with minor language editing and improvement of phrasing; literature selection, data extraction, interpretation, and conclusions were performed by the authors, who take full responsibility for the integrity and accuracy of the work.

## 5. Conclusions

This narrative review highlights that AD is not only a skin-limited disease, but a condition in which cutaneous and intestinal dysbiosis may coexist and potentially reinforce each other through gut–skin interactions and the NICE network. Early-life microbial colonization, delivery mode, and perinatal exposures appear to “prime” this axis and may explain later AD phenotypes.

Mechanistically, microbial metabolites (SCFAs, tryptophan-derived AHR ligands), GPCR signaling (FFAR2/GPR43, FFAR3/GPR41), and stress-driven HPA activation provide a plausible link between gut imbalance and cutaneous inflammation. On this background, microbiome-targeted interventions, particularly multi-strain, *Lactobacillus*-dominant probiotic formulations, have shown modest but relatively consistent clinical benefits, mainly in pediatric AD, and were generally well tolerated in the studied populations; however, follow-up was typically short and adverse-event ascertainment/reporting varied across trials, so long-term safety and safety in under-represented groups cannot be robustly determined.

However, the current evidence is heterogeneous (strains, doses, duration, outcomes) and mostly short-term, so probiotics are best regarded as adjuncts to standard dermatologic therapy, not replacements. Better strain-level reporting, parallel gut–skin profiling, age-adapted regimens, and long-term follow-up are needed before microbiome modulation can be routinely integrated into AD management.

In addition, because AD skin often shows reduced microbial diversity, loss of protective staphylococci, and overgrowth of *S. aureus*, microbiome modulation can be conceptualized as a dual strategy: systemic (oral probiotics targeting gut dysbiosis) plus local (microbiome-friendly emollients, topical/postbiotic formulations, and, when indicated, *S. aureus* decolonization). Future trials will need to report parallel gut–skin microbiome outcomes and barrier markers to capture the full effect of such combined approaches.

Finally, this narrative review is not intended as a clinical guideline; clinical decisions should be individualized and informed by patient context and the totality of evidence.

## Figures and Tables

**Table 1:** Randomized controlled trials (RCTs) evaluating microbiome-targeted interventions (probiotic/synbiotic, including one non-viable preparation) in atopic dermatitis (AD), published 2018–2025, identified through database searches and reference screening. Data were extracted from the primary RCT reports and, when available, their Supplementary Materials. NR indicates information not reported in the primary publication/supplements. For non-viable preparations (e.g., heat-killed products), CFU is not applicable and dosing is reported as grams/day as provided by the trial report. **Abbreviations:** AD, atopic dermatitis; CFU, colony-forming units; SCORAD, Scoring Atopic Dermatitis. Arrows indicate direction of change (↑ increase; ↓ decrease).

Author, Year, Country	Study Design	Population	Intervention Strain and CFU	Duration	Comparator	Outcome
So Hyun Ahn et al., 2020, Korea [45]	double-blinded, placebo-controlled, randomized study	Children aged 2–13 years with AD	*L. pentosus* DOSE: NR	12 weeks	placebo	Decrease in SCORAD
Angela MICHELOTTI et al., 2021, Italy [46]	randomized controlled trial (RCT)	80 adults with mild-to-severe AD Age: NR	mixture of lactobacilli (*L. plantarum* PBS067, *L. reuteri* PBS072 and *L. rhamnosus* LRH020) DOSE: *L. plantarum*—1 × 10^9^ CFU/daily *L. reuteri*—1 × 10^9^ CFU/daily *L. rhamnosus*—1 × 10^9^ CFU/daily	56 days	Placebo	improvement in skin smoothness, skin moisturization, self-perception; decrease in SCORAD index as well as in the levels of inflammatory markers associated with AD
Laura Carucci et al., 2022, Italy [47]	Randomized double-blind, controlled trial	patients aged 6–36 months with AD	*Lacticaseibacillus rhamnosus* GG Dose: 1 × 10^10^ CFU/daily	12 weeks	placebo	Decrease in SCORAD and DLQI
Marta Feíto-Rodríguez et al., 2023, Spain [48]	This double-blind, randomized, placebo-controlled clinical trial	70 participants with AD aged 4–17 years	probiotic mixture of *Bifidobacterium lactis*, *Bifidobacterium longum* and *Lactobacillus casei* Dose: 1 × 10^9^ CFU/daily	12 weeks	placebo	Decrease in SCORAD
Enza D’Auria et al., 2020, Italy [49]	A randomized, double-blind, placebo-controlled trial	Infants with moderate to severe atopic dermatitis, aged 6–36 months	heat-killed *Lactobacillus paracasei* CBA L74 (fermented rice flour) Dose: 8 g daily (CFU not applicable)	12 weeks	placebo	Decrease in SCORAD
Irfan A. Rather et al., 2020, South Korea [50]	Randomized Double-Blind, and Placebo-Controlled Study	children and adolescents (aged 3–18) with AD	*L. sakei* proBio65 live and dead cells Dose: 1 × 10^10^ CFU/daily	12 weeks	placebo	Decrease in SCORAD
Paula Danielle Santa Maria Albuquerque de Andrade et al., 2022, Brazil [51]	double-blind, randomized, placebo-controlled clinical trial	60 patients aged between 6 months and 19 years with mild, moderate, or severe AD	*Lactobacillus rhamnosus* HN001: 1 × 10^9^ CFU/daily *Lactobacillus acidophilus* NCFM: 1 × 10^9^ CFU/daily *Lactobacillus paracasei* Lcp-37: 1 × 10^9^ CFU/daily *Bifidobacterium lactis* HN019: 1 × 10^9^ CFU/daily	6 months	placebo	Decrease in SCORAD
C R S Prakoeswa et al., 2022, Indonesia [52]	randomized double-blind placebo-controlled trial	30 adults with mild and moderate AD	*Lactobacillus plantarum* IS-10506 Dose: 2 × 10^10^ CFU/ daily	8 weeks	placebo	SCORAD ↓ IL-4 ↓ IL-17 ↓ IFN-γ ↑ Foxp3+ ↑
Vicente Navarro-López et al., 2018, Spain [53]	double-blind, placebo-controlled intervention trial	children aged 4–17 years with moderate AD	*Bifidobacterium lactis* CECT 8145, *B longum* CECT 7347, and *Lactobacillus casei* CECT *9104* Dose: 1 × 10^9^ CFU/daily	12 weeks	Placebo	Decrease in SCORAD; fewer patient-days with topical corticosteroid use for flares
Richa Sharma et al., 2022, India [54]	randomized controlled study	114 children with AD	*Bacillus clausii* Dose: 4 × 10^9^ CFU/daily	12 weeks	placebo	SCORAD: no difference IL-17A: no difference; no correlation with severity

## Data Availability

No new data were created or analyzed in this study. Data sharing is not applicable to this article.

## Supplementary Materials

The following supporting information can be downloaded at: https://www.mdpi.com/article/10.3390/ijms27010365/s1.

## Abbreviations

The following abbreviations are used in this manuscript:

AD	Atopic dermatitis
AHR	Aryl hydrocarbon receptor
AMP	Antimicrobial peptide
CFU	Colony-forming unit
CRH	Corticotropin-releasing hormone
EASI	Eczema Area and Severity Index
FFAR2/GPR43	Free fatty acid receptor 2/G protein-coupled receptor 43
FFAR3/GPR41	Free fatty acid receptor 3/G protein-coupled receptor 41
FOXP3	Forkhead box P3
GABA	Gamma-aminobutyric acid
GPCR	G protein-coupled receptor
H_2_O_2_	Hydrogen peroxide
HDAC	Histone deacetylase
HO-1	Heme oxygenase-1
HPA	Hypothalamic–pituitary–adrenal
IBD	Inflammatory bowel disease
IBS	Irritable bowel syndrome
IL-4, -13, -31, -6, -1β, -10	Interleukin-4, -13, -31, -6, -1β, -10
MMP	Matrix metalloproteinase (e.g., MMP-1, MMP-9)
NICE	Neuro-immuno-cutaneous-endocrine
NLRP3	NLR family pyrin domain containing 3
Nrf2	Nuclear factor erythroid 2-related factor 2
PAMP	Pathogen-associated molecular pattern
RCT	Randomized controlled trial
SCFA	Short-chain fatty acid
SCORAD	Scoring Atopic Dermatitis
TEWL	Transepidermal water loss
Th2	T-helper 2 cell
Th17	T-helper 17 cell
TLR	Toll-like receptor
TNF-α	Tumor necrosis factor alpha
Treg	Regulatory T cell
UV	Ultraviolet
