# Supplementary material for: From Gut Dysbiosis to Skin Inflammation in Atopic Dermatitis: Probiotics and the Gut–Skin Axis—Clinical Outcomes and Microbiome Implications
Source: Int J Mol Sci. 2025 Dec 29;27(1):365. doi: 10.3390/ijms27010365 (PMC12785343; doi:10.3390/ijms27010365)
Supplement: Supplementary file 1 [file ijms-27-00365-s001.zip › Supplementary table S1 - ijms-4022118.pdf]

Database	Search query	Limits / filters	Last run + records
PubMed	(("atopic dermatitis" [Title/Abstract] OR eczema[Title/Abstract]) AND ("gut-skin axis" [Title/Abstract] OR "skin microbiome" [Title/Abstract] OR "cutaneous dysbiosis" [Title/Abstract] OR dysbiosis[Title/Abstract] OR "intestinal dysbiosis" [Title/Abstract] OR "gut microbiome" [Title/Abstract] OR "intestinal microbiome" [Title/Abstract]) AND (probiotic*[Title/Abstract] OR synbiotic* [Title/Abstract] OR postbiotic*[Title/Abstract] OR "microbiome modulation" [Title/Abstract]))	Humans; English; 2018/01/01-2025/10/31	Last run: Dec 2025; Records: n=157
Scopus	TITLE-ABS-KEY (("atopic dermatitis" OR eczema) AND (probiotic* OR synbiotic* OR postbiotic* OR "microbiome modulation") AND (randomi?ed OR placebo OR "double blind" OR "double-blind" OR trial OR RCT)) AND PUBYEAR > 2017 AND PUBYEAR < 2026	English; Article/Review; 2018-2025	Last run: Dec 2025; Records: n=322
Google Scholar	“atopic dermatitis” probiotic randomized trial SCORAD	custom range 2018-2025; results sorted by relevance; first 10 pages (first 100 results) screened manually	Last run: Dec 2025; Saved: n=14

Table S1. Database search strategy (last run: December 2025; coverage: 1 Jan 2018-31 Oct 2025)

1. PubMed search was performed using free-text keywords in Title/Abstract fields only; MeSH mapping was not applied;
2. Scopus query was intentionally RCT-focused to support identification of clinical trials for the RCT evidence table;
3. Date and document-type limits were applied via Scopus interface filters; the exported query string includes the corresponding PUBYEAR limits as displayed by Scopus;
4. Google Scholar results were screened manually by the author (first 10 pages/first 100 results, sorted by relevance) without exporting/importing records; duplicates were checked during screening and none were noted within the screened set.
