# Supplementary figures and images for: From Gut Dysbiosis to Skin Inflammation in Atopic Dermatitis: Probiotics and the Gut–Skin Axis—Clinical Outcomes and Microbiome Implications
Source: Int J Mol Sci. 2025 Dec 29;27(1):365. doi: 10.3390/ijms27010365 (PMC12785343; doi:10.3390/ijms27010365)

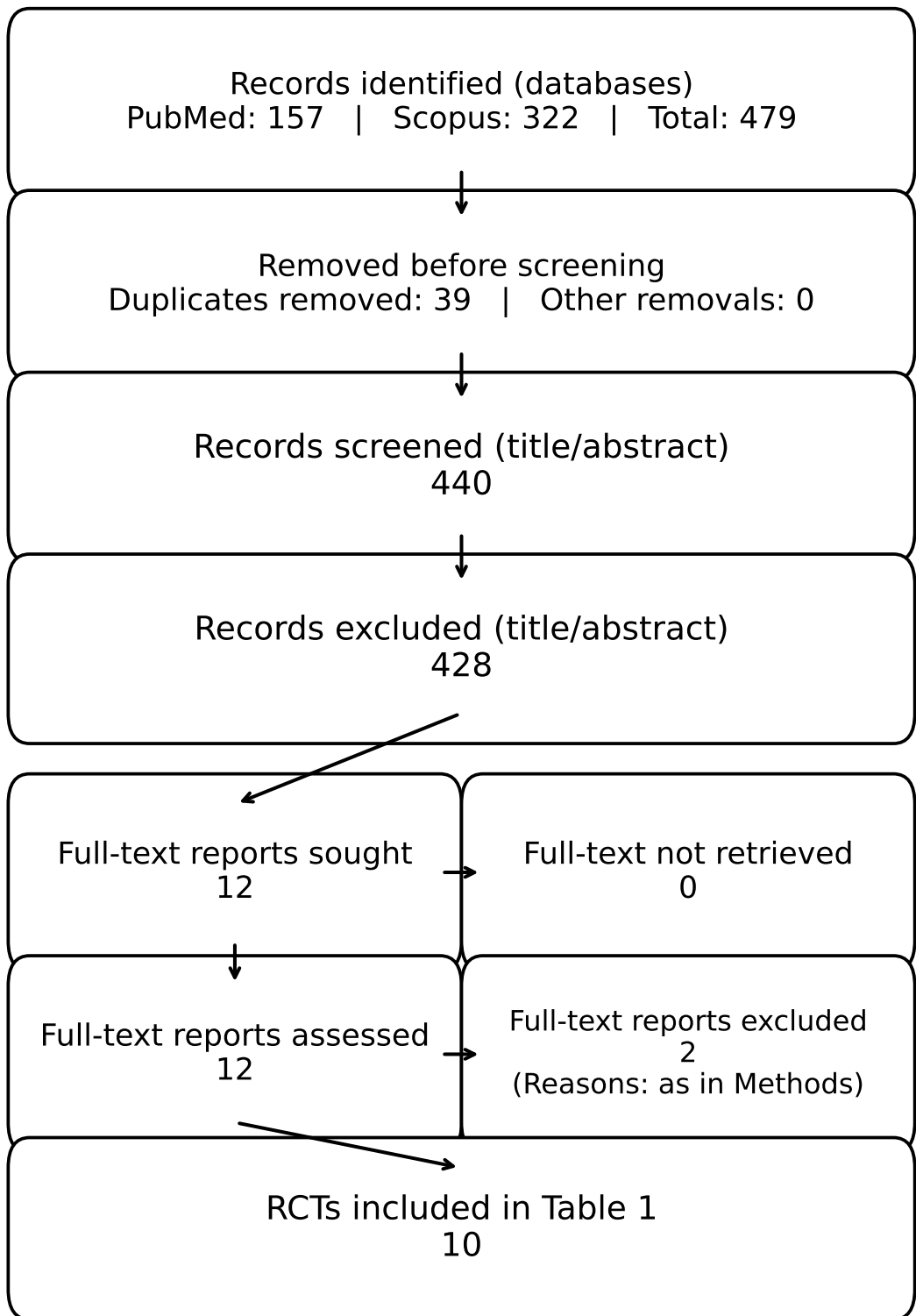

Note: Google Scholar (first 100 results screened; 14 retained) was used for cross-checking only.

Supplement: Supplementary file 1 [file ijms-27-00365-s001.zip › Figure S1. PRISMA-style flow diagram (adapted) for the RCT-focused search used to compile Table 1 - ijms-4022118.pdf]
